# Cationic Dendrimer G2-S16 Inhibits Herpes Simplex Type 2 Infection and Protects Mice Vaginal Microbiome

**DOI:** 10.3390/pharmaceutics12060515

**Published:** 2020-06-04

**Authors:** Carlos Guerrero-Beltrán, Inmaculada Garcia-Heredia, Rafael Ceña-Diez, Ignacio Rodriguez-Izquierdo, María Jesús Serramía, Francisco Martinez-Hernandez, Mónica Lluesma-Gomez, Manuel Martinez-Garcia, María Ángeles Muñoz-Fernández

**Affiliations:** 1 Immunology Section, Head Inmuno-Biology Molecular Laboratoy, Hospital General Universitario Gregorio Marañón, Instituto de Investigación Sanitaria Gregorio Marañón (IiSGM), Spanish HIV HGM BioBank, C/Dr. Esquerdo 46, 28007 Madrid, Spain; carlosguerrero_1992@hotmail.com (C.G.-B.); rcena48@gmail.com (R.C.-D.); igna.iri.93@gmail.com (I.R.-I.); mariajesus.serramia@salud.madrid.org (M.J.S.); 2 Department of Physiology, Genetics, and Microbiology, University of Alicante, C/San Vicente s/n, 03080 Alicante, Spain; inma.garcia@ua.es (I.G.-H.); franmh@ua.es (F.M.-H.); m.lluesma@ua.es (M.L.-G.); 3 Networking Research Center on Bioengineering, Biomaterials and Nanomedicine (CIBER-BBN), 28029 Madrid, Spain

**Keywords:** G2-S16 polyanionic carbosilane dendrimer, HSV-2, semen, microbicide, female mice, vaginal microbiome, HCV

## Abstract

The G2-S16 polyanionic carbosilane dendrimer is a promising microbicide that inhibits HSV-2 infection in vitro and in vivo in mice models. This G2-S16 dendrimer inhibits HSV-2 infection even in the presence of semen. Murine models, such as BALB/c female mice, are generally used to characterize host-pathogen interactions within the vaginal tract. However, the composition of endogenous vaginal flora remains largely undefined with modern microbiome analyses. It is important to note that the G2-S16 dendrimer does not change healthy mouse vaginal microbiome where *Pseudomonas* (10.2–79.1%) and *Janthinobacterium* (0.7–13%) are the more abundant genera. The HSV-2 vaginally infected female mice showed a significant microbiome alteration because an increase of *Staphylococcus* (up to 98.8%) and *Escherichia* (30.76%) levels were observed becoming these bacteria the predominant genera. BALB/c female mice vaginally-treated with the G2-S16 dendrimer and infected with the HSV-2 maintained a healthy vaginal microbiome similar to uninfected female mice. Summarizing, the G2-S16 polyanionic carbosilane dendrimer inhibits the HSV-2 infection in the presence of semen and prevents the alteration of mice female vaginal microbiome.

## 1. Introduction

A safe and effective microbicide to prevent sexual transmission infections (STIs) such as HSV-2 is still needed. It is important to note that microbicides that have been proven to inhibit STIs had failed in different clinical trials [1,2], most probably because these microbicides failed to prevent semen-exposed infection [3] as well as they interfere with the microbiota of healthy individuals, increasing the HSV-2 infection or generating bacterial vaginosis (BV). Recently, the relationship between BV and the efficacy of antiretroviral-based prevention technologies, especially microbicides topically applied to the female vaginal surface have been a focus of attention. Klatt et al. revealed that not only the lack of adherence was the cause of the impaired efficacy of the polymerase inhibitor tenofovir (TFV) gel microbicide in the CAPRISA 004 clinical trial, but also the BV modulated the efficacy due to the drug depletion via bacterial metabolism [4,5]. Not only TFV but also many other microbicide candidates failed due to the microbiome impairment that may lead to the BV and subsequent increasing the risk of STIs. Therefore, it is urgently needed to research the relationship between leading microbicides and changes in vaginal microbiome structure and diversity.

The healthy vaginal microbiome consists of a wide variety of bacterial species that maintains an acidic pH by hydrogen peroxide and lactic acid production [6]. Alterations in this ecosystem cause several vaginal infections, such as BV, a symptomatic clinical disease distinguished by a low abundance of *Lactobacillus* sp. accompanied by an overgrowth of anaerobic microorganisms [7], which is related to a noticeably increased risk for acquisition of STIs, including HIV-1 [8,9,10] and HSV-2 [11,12]. The higher risk could be explained through multiple mechanisms: increasing inflammation and recruiting of target cells, epithelial barrier disruption, and wound-healing impairment that overall have toxic effects and modify not only the healthy vaginal microbiome but also the structural integrity of the vaginal mucosal epithelium. The maintaining of a healthy vaginal microbiome is an important biological factor that protects from pathogenic microorganisms [13].

Nanotechnology provides new and suitable approaches to obtain novel, potent, and safer antiviral agents, such as dendrimers [14]. Our group has shown that one of the most promising microbicides could be the G2-S16 polyanionic carbosilane dendrimer [15,16]. The potent activity of this G2-S16 dendrimer against HIV-1 and HSV-2 has been demonstrated [17,18,19]. The G2-S16 dendrimer is a second-generation dendrimer with a carbosilane structure containing 16 sulfonate peripheral groups derived from a silicon core [16]. We have selected this G2-S16 dendrimer as a possible new vaginal topical microbicide based on its short reaction times, wide availability of reagents, high reproducibility, and quantitative yields of reaction. The specific design and the three-dimensional structure of the G2-S16 dendrimer are essential features to interfere with envelope proteins of HIV-1 or HSV-2 and receptors on the host cells, conferring a multifactorial and nonspecific ability. Many in vitro and in vivo studies confirmed the safety and efficacy of this G2-S16 dendrimer to prevent HSV-2 and HIV-1 infections [17,19]. The G2-S16 dendrimer was stable at various pHs and in the presence of seminal fluids maintaining the anti-HIV-1 activity overtime, this dendrimer did not generate any type of drug resistance and did not cause inflammation or irritation in the vaginal mucosa after administration of G2-S16 dendrimer at various concentrations and different times in female mice and rabbits [17,19,20]. When BALB/c mice or humanize (h)-BLT mice were pre-treated with topical 3% G2-S16 dendrimer and then female vaginally exposed to R5-HIV-1_JR-CSF_ or HSV-2 333, G2-S16 dendrimer efficiently prevented female vaginal HIV-1 transmission by 84% [19] and HSV-2 by 100% [17]. However, determine the role of G2-S16 dendrimer against HSV-2 infection in presence of semen must be studied since it has been reported that the presence of semen enhances HSV-2 infection in a 10 fold rate [21]. In the context of a vaginal microbicide, to assess if the G2-S16 dendrimer can implement all these characteristics without interfering with the normal vaginal microbiota is one the last step to achieve a proof of concept. In this sense, the female BALB/c model was set up to evaluate the effect of the G2-S16 dendrimer on the vaginal microbiome. To our knowledge, the impact of new nanoparticles as dendrimers on mice vaginal microbiome and the infection of HSV-2 have not been elucidated. A metagenomic approach was employed to characterize the mice vaginal microbiome, host-bacterial dysbiosis caused by HSV-2 infection and to analyze the effect of the G2-S16 polyanionic carbosilane dendrimer.

## 2. Methods

### 2.1. Dendrimer, Cell Culture Semen Samples, and Virus Strain

Polyanionic carbosilane dendrimer C_112_H_244_N_8_Na_16_O_48_S_16_Si_13_, named as G2-S16, was synthesized and tested by the Dendrimers for Biomedical Applications Group of University of Alcalá (Madrid, Spain) as previously described [16]. G2-S16 dendrimer present 16 sulfonate groups in the periphery, and a molecular weight 3717.15 g/mol.

African green monkey kidney Vero cell line (ATCC CCL-81, Manassas, VA, USA) was obtained from the American Type Culture Collection. Vero cells were grown in DMEM supplemented with 5% FBS containing 1% l-glutamine, and an antibiotic mix (125 µg/mL ampicillin, 125 µg/mL cloxacillin and 40 µg/mL gentamicin) (Sigma, St. Louis, MO, USA). Vero cells were cultivated in 5% CO_2_ at 37 °C.

Semen samples of healthy men donors were obtained after informed consent [22] as described before [15,17]. The semen samples were aliquots at −20 °C for further experiments.

HSV-2 strain 333 isolate (HSV-2) was expanded on Vero cells, titrated by plaque assay as PFU/mL and stored at −80 °C. Briefly, Vero cells were seeded in p24 well plates at 1.75 × 10^5^ cells/well and infected with serial dilutions of HSV-2 viral stock in 300 µL of DMEM 2% FBS. After 2 h, supernatants were removed and 300 µL of fresh DMEM 2% FBS with 0.4% of human immunoglobulin (IgG) were added. After 48 h, the medium was removed and Vero cells were stained with methylene blue (300 mg/L) for 1 h. Lysis plates were counted and the viral stock was titrated as PFU/mL.

### 2.2. Efficacy of G2-S16 Polyanionic Carbosilane Dendrimer against HSV-2 Infection in Presence of Semen

To assess the activity of G2-S16 polyanionic carbosilane dendrimer in presence of semen, Vero cells were seeded in 24-well plates at 1.75 × 10^5^ cells/well and expose to maximum non-toxic concentration for G2-S16 dendrimer (10 µM) [17]. HSV-2 was incubated for 10 min with semen (10%) or PBS as described previously [17,23]. After the semen/HSV-2 incubation, the Vero cells were infected with HSV-2/semen inoculum at MOI 0.001. The Vero cells were washed with PBS at 2 h post-infection to remove unabsorbed viruses. HSV-2 infection remained in DMEM containing 2% FCS and 0.4% IgG. After 48 h, the medium was removed, and Vero cells were stained with Methylene Blue 300 mg/L (Sigma, St. Louis, MO, USA) 1 h, and the lysis plaques were counted. Results were related to infection controls.

### 2.3. Experimental Mice In Vivo Design

Twelve female BALB/c mice of seven weeks old with an average weight of 20 ± 3 g were purchased (Charles River Laboratories, Wilmington, MA, USA). BALB/c mice were housed at the Centro Biologia Molecular Severo Ochoa (CBMSO). Animal studies were approved by Ethic Experimental Animals Committee of the Centro de Biología Molecular “Severo Ochoa” (EEACCBMSO Institutional Animal Care and Use Committee (EEAC-CBMSO, Madrid, Spain)). (PROES 136/14; Register number ES-280790000180). All experiments were carried out following EECA-CBMSO, National (Royal Decree 1201/2005) and the Directive 2010/63/EU of the European Parliament guidelines and regulations. The G2-S16 polyanionic carbosilane dendrimer was formulated as a water-based gel because gels are optimal formulations to ensure that the microbicide begins to act quickly. The vehicle is a hydroxyethyl-cellulose (HEC) gel and the active pharmaceutical ingredient is the G2-S16 dendrimer. Therefore, 3% weight/volume (*w/v*) of the G2-S16 dendrimer was mixed in 1% (*w/v*) of HEC which is biocompatible with normal human vagina [17,20,24].

**Figure 1:**
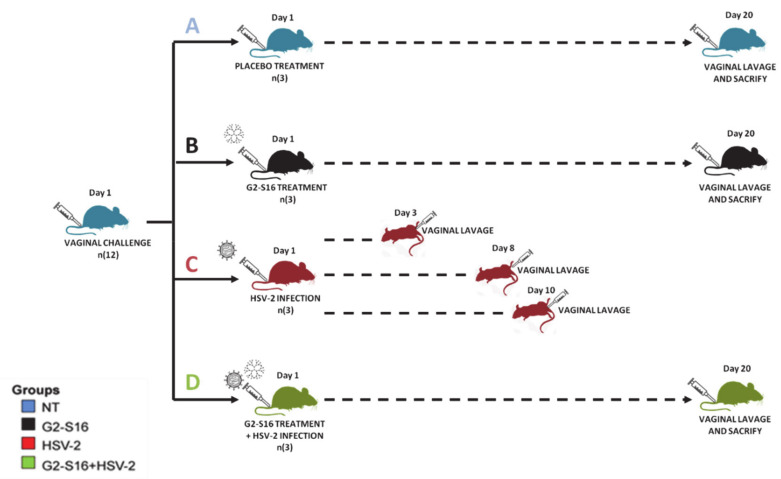
Schematic representation of experimental design and samples. Four mice groups (3 mice per group) were analyzed and vaginal lavages were collected at day 1 and day 20. Group NT (healthy untreated; samples (**A**), Group G2-S16 (treated with dendrimer; samples (**B**), Group HSV-2 (infected with HSV-2; samples **C**) that was sampled prior infection and post-infection, when a mouse reached a 4 diseases score (between days 3–10) and Group G2-S16+HSV-2 (treated with dendrimer and post-infected with HSV-2; samples **D**).

BALB/c female mice were randomized into four groups of only three mice per group: group control with no treatment (group NT), group treated with G2-S16 dendrimer (group G2-S16), group infected by HSV-2 (group HSV-2), and group treated with G2-S16 dendrimer and post-infected by HSV-2 (group G2-S16 + HSV-2) (Figure 1). Two vagina lavage samples were taken per female mouse. For all female mice, the first sample (named as “day 1,” Figure 1) corresponded to vagina lavages collected before any treatment and infection, which overall represents a healthy untreated vagina (NT, Figure 1). The second and last vagina lavage sample per female mouse was taken after the G2-S16 dendrimer treatment and/or the HSV-2 infection. In this case, for all groups, except for the group G2-S16 dendrimer, at 20 days treatment and/or post-infection, female mice were sacrificed and vaginal lavages were performed. Daily examination for body weight and genital pathology was performed over 20 days. Disease score was graded according to a 4-point scale: 0, no apparent infection; 1, genital erythema; 2, moderate genital infection; 3, purulent genital ulceration and hair loss, generally poor condition; and 4, severe ulceration of genital and surrounding tissue, and hind limb paralysis (leading to euthanasia) [25,26]. When HSV-2 infected female mice reached a 4 points grade, which occurred between day 3 and 10, they were sacrificed according to ethical statements, and subsequently, vaginal lavages were collected. Five days previous to vaginal the HSV-2 challenge and sample collection, the female mice from HSV-2 group and G2-S16+HSV-2 group were treated with a subcutaneous injection of 2 mg medroxyprogesterone acetate (Depo-Provera [depo]; Pfizer, New York, NY, USA) to increase susceptibility to HSV-2 infection [27]. The G2-S16 and G2-S16+HSV-2 female mice groups were treated vaginally with 30 µL of 1% HEC gel. One hour later, both female mice groups were infected with 10^5^ PFU/20 µL of HSV-2 and maintained in a supine position for 15 min. Vaginal lavages were collected as follows: female mice were anesthetized with isoflurane (2-chloro-2-(difluoromethoxy)-1,1,-trifluoro-ethane, Forane, Abbott, Perú) and then lavages were performed with 50 µL of sterile buffer PBS to recover the vaginal microbiome that was finally stored at −80 °C before the DNA extraction.

### 2.4. DNA Extraction, Microbiome Sequencing, and Sequence Analysis

DNA extraction from 50 µL of vaginal lavages was performed with DNA DNeasy Blood & Tissue Kit^®^ (Qiagen, ref. 69504) according to the manufacturer’s protocol for Gram-positive and negative bacteria. Negative control of sterile PBS was used as a blank for DNA extraction to assess the presence of DNA contaminants in reagents and treated like the rest of the samples. DNA was quantified by Qubit dsDNA HS Assay Kit (Life Technologies, ref. Q32851). Metagenomic libraries for DNA sequencing were prepared by using the Nextera XT DNA library (ref. FC-131-1024, Illumina) according to the manufacturer’s protocol. Microbial metagenomes were sequenced by Illumina technology in a NextSeq 500 sequencer (2 × 150, pair-end) in the Genomics Center of Foundation for the Promotion of Health and Biomedical Research in the Valencian Region (FISABIO, Valencia, Spain). The inclusion of controls was paramount to monitor the potential impact of DNA contamination from molecular reagents in microbial diversity assays [28]. Here, a blank that consisted of the same PBS buffer used for vaginal lavages was subjected to the same procedure as samples (e.g., DNA extraction). High sensitivity DNA measurements conducted by fluorometry indicated no detectable DNA in molecular reagents. Metagenomic library procedure with Nextera XT (see methods) failed for that blank sample, which indicates that the presence of exogenous bacterial DNA contaminants in our metagenome datasets from vaginal samples is likely negligent or significantly low.

Female mouse’s DNA belonging reads were removed according to BMTagger program [29], using as reference *Mus musculus* (NC_000064-NC_000087) and *Rattusnorvegicus* (NC_005100-NC_005120, NC_024475). We performed another thorough cleaning of the female mouse reads by comparing the resulting reads with the *nt* database from NCBI with *e*-value 0.00005 as a cut-off and those reads matching with any rodent genome were removed. Microbial reads were quality filtered using trimmomatic v 0.36 program [30] with the following parameters: phred33, leading:3, trailing:3, sliding window: 4:15, minlen:36. Annotation of reads was done comparing them with the NT database (NCBI), using 70% identity as a cut-off. K-mer based comparison between samples was done with MetaFast using at least 900 sequences longer than 100 bp of each sample [31]. The microbiome sequencing data were submitted to NCBI SRA and are available with the bioproject number PRJNA480951.

### 2.5. Statistical Analysis

Statistical analysis and Shannon index calculations were performed using R package Vegan. Alpha-diversity was done comparing the Shannon index for each sample using ANOVA (ANalysis Of VAriance). First, we check that our samples accomplish all the ANOVA requirements (regarding the samples, normalization of the levels, and variance homogeneity). A comparison of the microbial composition was performed by using two-way PERMANOVA (Permutational Multivariate Analysis of Variance Using Distance Matrices) to compare the abundances of the microbes in the samples. Moreover, to analyze the inter-group variability we calculated the multivariate homogeneity of the group’s dispersions (variances) with an ANOVA analysis to compare the variances of the dispersions and a Tukey test, which is calculated through pairwise comparison of all means to determine which are significantly different. Finally, to visualize similarities or dissimilarities of our data, a principal coordinated analysis was calculated, which is a statistical tool for reducing the dimensionality of large datasets, creating new uncorrelated variables (principal components) that successively maximize variance.

## 3. Results

### 3.1. Inhibition of G2-S16 Polyanionic Carbosilane Dendrimer in Presence of Semen

**Figure 2:**
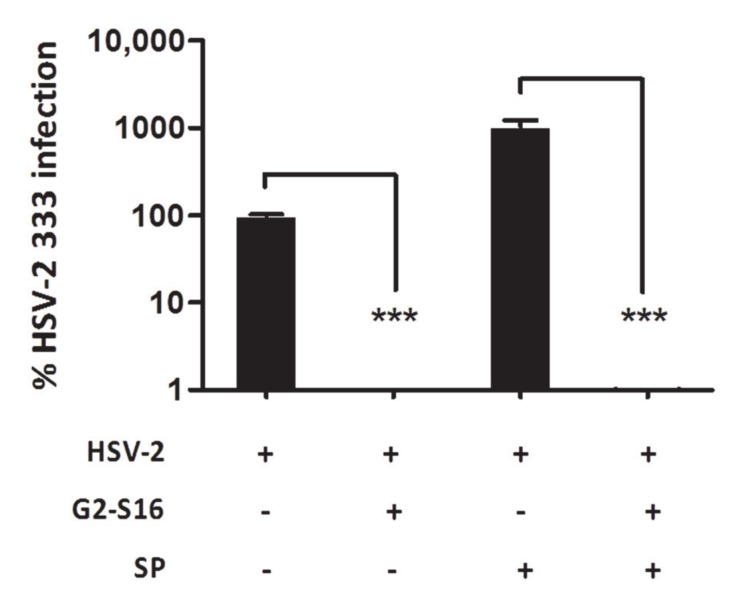
Inhibition of HSV-2 in the presence of semen. Vero cells were pretreated with a non-toxic concentration of G2-S16. After 1 h cells were infected with HSV-2 in the presence or absence of seminal plasma (SP). Data represent mean ± SD from three independent experiments by duplicate. ***: *p* < 0.001. Infection was revealed by plaque reduction assay. HSV-2 infection at MOI 0.001 was used as infection control, and data were related to control. +/-: Presence or absence of treatment.

It had been widely described that the presence of semen enhances the HSV-2 infection [21,32] as well as other sexually transmitted viruses, such as HIV-1 [33,34]. In that sense, reports have demonstrated that exposure of HSV-2 to concentrations of seminal plasma increased by up to 30 fold the HSV-2 infection [21]. To analyze the efficacy of the G2-S16 dendrimer in the presence of seminal plasma, HSV-2 was incubated with 10% of SP, and after 10 min Vero cells were infected (Figure 2).

The presence of SP increased the HSV-2 infection in a magnitude order (Figure 2). However, the G2-S16 dendrimer was able to inhibit completely the HSV-2 infection in normal conditions (Figure 2). Interestingly, when HSV-2 was incubated with SP and treated with G2-S16 dendrimer, this dendrimer inhibited increased infection with the same behavior, which in the absence of semen, inhibited >99% of HSV-2 infection (Figure 2). Our data clearly showed that though the semen potentiates the HSV-2 infection up to a 10-fold increase, the G2-S16 dendrimer was capable to inhibit completely the HSV-2 infection >99%.

### 3.2. Experimental Design and Vaginal Microbiome Analyses

The ability of vaginal bacteria to manipulate mucosal immunity and barrier properties has the potential to enhance susceptibility to HSV-2 infection, primarily through causing a disturbance in the vaginal microbiome [17]. We studied whether the G2-S16 dendrimer causes any adverse effect on a healthy vaginal microbiome in BALB/c female mice (Figure 1). The vaginal microbiome of 24 samples from 12 BALB/c female mice was analyzed to get some biological insights into the microbiome composition and diversity of healthy BALB/c female mice vagina and the impact of the G2-S16 dendrimer on vaginal microbiome under healthy and HSV-2 infection conditions (Figure 1). In this preliminary proof of concept, twelve BALB/c female mice were randomized into four groups of only three mice per group (Figure 1). The first NT group, control female mice without any treatment. In the second G2-S16 dendrimer group, female mice treated vaginally with 1% HEC gel with only the G2-S16 dendrimer. In the third HSV-2 group, female mice vaginally infected with the HSV-2. The fourth group, female mice treated with G2-S16 dendrimer and HSV-2 with 1% HEC gel with the G2-S16+HSV-2 infected. According to the experimental design, a total of fifteen vaginal samples were obtained to analyze the normal vaginal microbiome of healthy untreated BALB/c female mice (group NT, Figure 1).

### 3.3. Microbial Community of Healthy Female Mice Vagina

**Figure 3:**
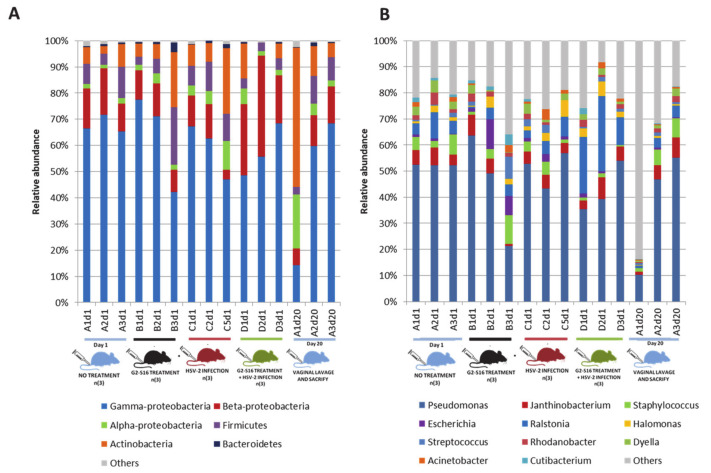
Composition of the vaginal microbiome in healthy mice (group NT). Relative abundances at (**A**) phylum level and (**B**) genus level. The relative taxonomic abundances were calculated from metagenomic data. Taxonomy was assigned when reads showed ≥70% identity against subjects in the nucleotide (nt) database (NCBI). Coding color and nomenclature of samples analyzed by metagenomics are those used in Figure 2: capital letter “A–D” refers to the type of samples (A = healthy untreated; B = treated with dendrimer; C = infected with HSV-2; D = treated with dendrimer and post-infected with HSV-2), number “n” refers to replicate samples, and “d-number” refers to sacrificed day.

The microbial diversity of healthy female mice vagina was determined from samples belonging to the NT group (Figure 3). From each BALB/c female mouse, a vaginal lavage with 50 µL of sterile PBS was performed [35] to obtain vaginal microbiome samples that were further subjected to DNA extraction and metagenomic sequencing by Illumina technology (Supplementary Table S1). After the bioinformatic cleaning process that remove BALB/c female mouse DNA sequences in datasets, the remaining reads were compared by BLASTn program against “nt” database of Gen bank, and bacterial reads were taxonomically assigned by using a cutoff of 70% identity (Supplementary Table S2; see details in methods). The most abundant phylum in nearly all of the healthy untreated samples was Proteobacteria, being Gamma proteobacteria the most abundant in BALB/c female mice vagina (14.26–77.4%). Moreover, Actinobacteria (2.88–53.23%), Firmicutes (2.88–22.05%), and Bacteroidetes (0.21–3.67%) phyla were also detected in 93.75% of the NT group (Figure 3A). In addition, Proteobacteria, Alpha- (1.26–20.64%) and Beta-proteobacteria (3.68–38.5%) were also present in all samples studied. The composition of the vaginal microbiome revealed a clear dominance of *Pseudomonas* (10.2–63.7%). However, other bacteria such as *Ralstonia* (0.48–28.6%), *Escherichia* (0.33–11.3%), *Janthinobacterium* (0.7–8.1%), *Staphylococcus* (0.5–11%), or *Streptococcus* (0.42–8.45%) was also found, although with a low prevalence (Figure 3B). Another vaginal sample from a BALB/c female mouse taken five months before and independently from the BALB/c mice group NT (sample R0) showed similar microbial diversity (Supplementary Figure S1), which indicated that vaginal microbiome data from the BALB/c female mice group NT were consistent and stable through the time.

### 3.4. Variability of Microbial Composition in HSV-2 Infection

**Figure 4:**
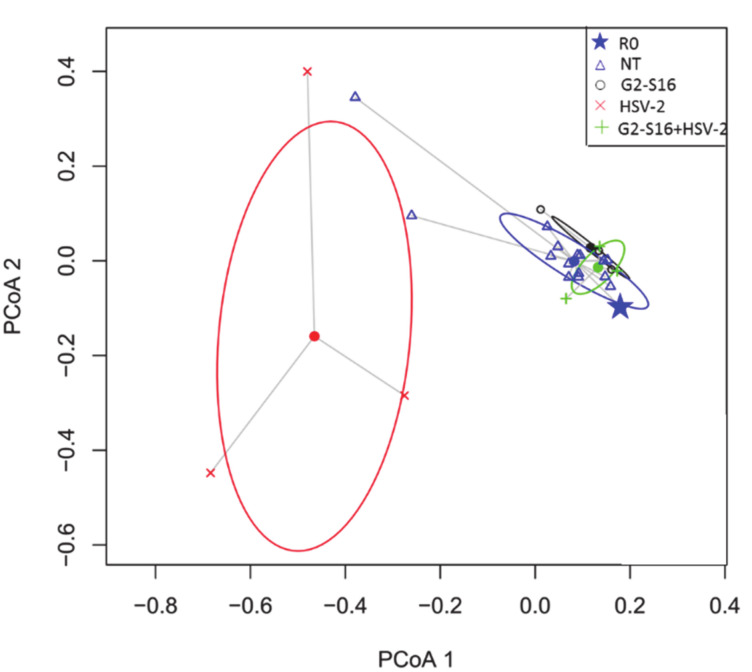
Principal Coordinate Analysis (PCoA). Analysis of multivariate homogeneity of group dispersions (variances) comparing Bray–Curtis distances. The figure shows the major PCo1 and PCo2. Ellipses show the standard deviation. Filled dots represent the median of the values in each group. Group NT or healthy mice, Sample “R0” (see Supplementary Figure S1) is marked with a star. Group G2-S16 or mice treated with G2-S16 dendrimer. Group HSV-2 or mice infected with HSV-2 and, group G2-S16+HSV-2 or mice treated with G2-S16 dendrimer and infected with HSV-2. Further explanation can be found in the text.

The HSV-2 infection could cause a microbial dysbiosis that compromises the vagina health status [36]. A significant difference in the microbial composition of NT group, G2-S16 group, HSV-2 group, and G2-S16+HSV-2 group analyzed by the Permanova statistical was showed (Supplementary Table S3). The dissimilarity of microbial composition among samples was estimated by Bray–Curtis distance, which is a statistical tool used to quantify the differences in species populations between two different sites [37]. ANOVA analysis of multivariate homogeneity of group dispersions (variances) was used as a measure of beta diversity [38], also exerted significant differences among the NT, G2-S16, HSV-2, and G2-S16+HSV-2 four groups (Supplementary Table S4). These differences were statistically significant among the microbiome of the HSV-2 group in comparison with the G2-S16 group and G2-S16+HSV-2 group by using the Tukey comparison method. Interestingly, when comparing the G2-S16+HSV-2 group with the NT group, there were no significant differences (*p*-value: 0.08). The principal coordinate analysis (PCoA) highlighted changes in microbial diversity in HSV-2 infected mice (Figure 4). Furthermore, in microbial diversity and composition among the BALB/c female mice HSV-2 group showed higher heterogeneity in comparison with the rest of NT, G2-S16, and G2-S16+HSV-2 groups, because no substantial shift in microbial composition among NT, G2-S16, and G2-S16+HSV-2 groups were found. Our data confirmed that HSV-2 infection induced a significant shift regarding the microbial composition of BALB/c female mice vagina. In contrast to group NT, the vagina composition of BALB/c female mice group HSV-2 was dominated by *Staphylococcus* (up to 98.8% in sample C1d8) as dominant genus, *Escherichia* (30.76%) and *Acinetobacter* (30.65%) (Figure 4). The nearest *Staphylococcus* species was *S. sciuri.* We were able to recover and assemble the full genome of *S. sciuri* from C1d8 sample (Supplementary Figure S2).

### 3.5. Effect of the G2-S16 Polyanionic Carbosilane Dendrimer on Microbial Diversity

**Figure 5:**
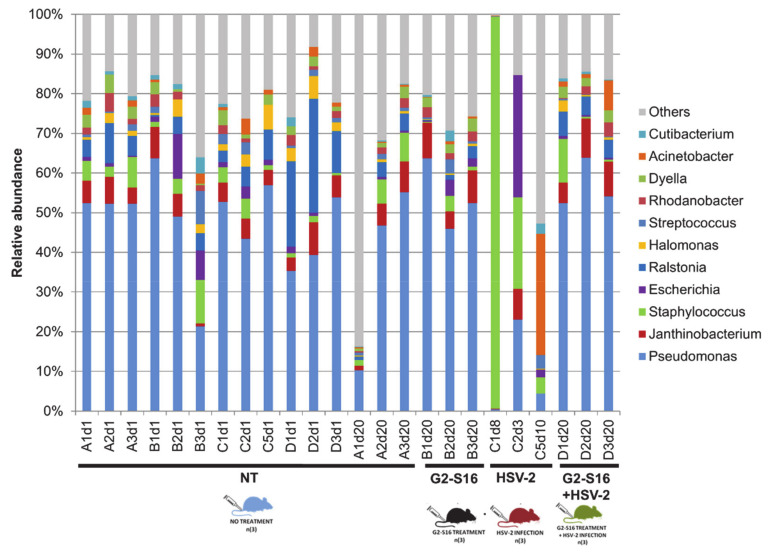
Comparison of vaginal microbiomes at the genus level. Relative abundances comparing the reads to nucleotide (nt) database (NCBI). Samples are clustered in groups NT, G2-S16, HSV-2, and G2-S16+HSV-2. Group NT consisted of control mice without any treatment. Group G2-S16 consisted of mice treated vaginally with 1% HEC gel and G2-S16 dendrimer as an active ingredient. Group HSV-2 consisted of mice HSV-2 infected, and group G2-S16+HSV-2 consisted of mice treated with 1% HEC gel with G2-S16 and infected with HSV-2. Coding color and nomenclature of samples analyzed by metagenomics are those used in Figure 2 and Figure 3: capital letter “A–D” refers to the type of samples (A = healthy untreated or NT; B = treated with dendrimer or G2-S16; C = infected with HSV-2; D = treated with dendrimer and post-infected with HSV-2), number “n” refers to replicate samples, and “d-number” refers to sacrificed day.

**Figure 6:**
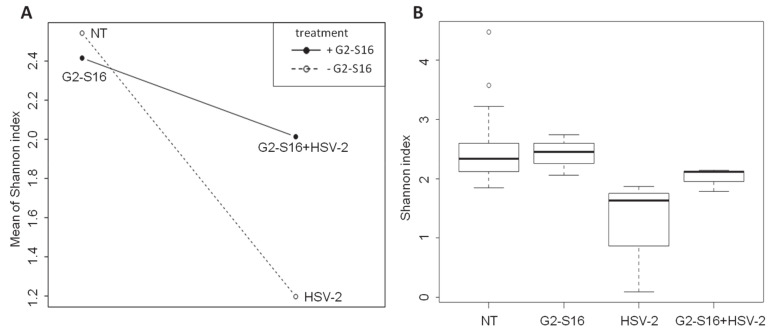
Comparison of vaginal microbiome diversity within groups of studied mice. Diversity is expressed based on the Shannon Index. (**A**) Interaction plot showing the Shannon index mean relation between groups using as independent factor HSV-2- infected group (group HSV-2) and treated mice with G2-S16 dendrimer and with HSV-2 (group G2-S16+HSV-2). (**B**) Boxplot representing Shannon index means for different groups and the maximum and minimum values showing data distribution within groups.

As shown in Figure 5, the microbiome composition of NT group and G2-S16 treated group were similar and clustered together. Furthermore, variation in the Shannon diversity Index was not observed among BALB/c mice vaginal samples from NT group and G2-S16 treated group (Figure 5). Moreover, the BALB/c female mice from the G2-S16+HSV-2 group showed minor changes in microbial diversity in comparison with the HSV-2 group (Figure 5 and Figure 6A). The Shannon index boxplot showed that the mean values of the G2-S16+HSV-2 group were very close to the NT group and G2-S16 group (Figure 6A). Group HSV-2 exerted the lowest Shannon index value (Figure 6B). Changes in microbial diversity were also detected during the BALB/c mice vaginal HSV-2 infection (Figure 6) since the vaginal microbiome of HSV-2 infected BALB/c mice showed the lowest values of Shannon diversity index (Figure 6B).

ANOVA analysis of the Shannon index showed significant differences between NT group and HSV-2 group (Figure 6B, Supplementary Table S5), while no significant differences were observed between NT group with those BALB/c female mice from G2-S16 group (Figure 6B). As depicted in Figure 6, PCoA plot data and ANOVA of beta dispersion indicated that no significant differences were found in microbial composition among NT, G2-S16, and G2-S16+HSV-2 groups.

## 4. Discussion

The composition of the vaginal microbiome is crucial for the prevention of STIs [39,40]. In that sense, to evaluate the proper function of potential candidate microbicides is one of the major goals in microbicide development. As many microbicide candidates failed due to vaginal dysbiosis, to determine the role of the microbicides on the vaginal microbiome is needed [41]. A safe and potent microbicide must inhibit not only HSV-2, but also other STIs, such as HIV-1, cytomegalovirus, HCV, even in presence of semen, which is known to increase viral infections, and on the other hand, must be innocuous for normal vaginal microbiota. To achieve this knowledge, BALB/c female mice models provide novel and useful information, which can be used in the first step for human clinical trials. Once we demonstrated the efficacy of the G2-S16 dendrimer against HSV-2 infection even in the presence of seminal plasma, a metagenomic approach was carried out in BALB/c mice. Despite the inter-individual variability that has an important influence on the microbiome analysis [28], some common microbial diversity and structure patterns were found at phylum and genus levels in NT BALB/c female mice samples. We did not monitor the stage of the estrous cycle at the time of sampling, which could change the bacterial profile of the vagina in animals and/or humans [42,43]. Data revealed that the most abundant phylum was Proteobacteria, with the group Gammaproteobacteria as the most prevalent (14.26–77.4%) (Figure 3A). At the genus level, we observed a clear dominance of *Pseudomonas* (10.2–63.7%). These results highlighted the similarity of the vaginal and lung microbiome as Barfod et al. revealed that the pulmonic and vaginal community shared more than 100 genera [35]. It is well known that the vaginal microbiome of BALB/c female mice differs from that vaginal microbiome of women [44]. In non-human mammals and those mammals with near-neutral vaginal pH [45], as here with BALB/c mice, the presence of *Lactobacillus* spp. is nearly undetectable, while other bacterial species dominate vaginal microbiomes, such as *Corynebacterium* spp. in guinea pig [46], *Escherichia* in giant pandas, or *Aggregatibacter* spp. in cows [44]. At genus-level resolution, each animal tends to have a unique vaginal microbiome composition with a limited degree of overlapping. In humans, the lack of a *Lactobacillus*-dominant vaginal environment was identified as an important risk factor not only for HSV-2 infection but also for HIV-1 infection [7,13,47]. Reciprocally, HIV-1 seropositive women were more likely to have episodes of BV [48]. In this study, we report a vaginal microbiome shift in HSV-2-infected BALB/c female mice, showing that the dominant genus was *Staphylococcus,* followed by *Escherichia* and *Acinetobacter,* in contrast to healthy BALB/c female mice, which vaginal microbiome was dominated by Proteobacteria. It has been previously shown that *Staphylococcus sciuri* can be pathogenic not only in humans but also in animals [49]. We speculate that some of these deep changes in the microbiome, such as that observed for the full dominance of *Staphylococcus* could be a bacterial post-infection triggered by HSV-2 dysbiosis.

The differences in beta bacterial diversity data were depicted in a principal coordinate analysis (PCoA) that represents the sample distances from statistical analysis into a visually manageable two-dimensional scheme. Significant changes and heterogeneity in microbial diversity in HSV-2 infected BALB/c mice were observed. These results are in concordance with the strong bacterial dysbiosis in HSV-2 infected women [12,50]. Interestingly enough, many drugs vaginally applied have a negative effect on the local microbiome, thus inducing a severe BV. We aimed to prove that the leading candidate for microbicide G2-S16 dendrimer not only does not affect a healthy vaginal microbiome but also this dendrimer protects vaginal microbiome when a BALB/c mouse is HSV-2 infected.

The composition of the vaginal microbiome of healthy BALB/c mice was similar to those BALB/c mice treated with G2-S16 and no variation of Shannon diversity index was observed. Our results indicate that the vaginal application of G2-S16 dendrimer does not modify or induce a shift in BALB/c mice vaginal microbiome. Furthermore, BALB/c mice treated with the G2-S16 dendrimer and infected with HSV-2 (G2-S16+HSV-2) exerted a composition very similar to healthy BALB/c mice.

Figure 3 showed no significant differences in microbial composition between BALB/c mice groups NT, G2-S16, and G2-S16+HSV-2 by PCoA plot data and ANOVA of beta dispersion. Our results suggest a protective role of the G2-S16 dendrimer in the context of the HSV-2 infection, not only preventing viral entry but also maintaining vaginal microbiome composition similar to that of healthy BALB/c mice. Many studies prove the safety and efficacy of the G2-S16 dendrimer in vivo [15,17,19]. Our work represents a paramount step further in a new context that supports subsequent clinical trials to assess its safety in women.

Summing up, we have demonstrated that the G2-S16 dendrimer does not alter the BALB/c healthy vaginal microbiome and this G2-S16 dendrimer protects the microbiome in the presence of HSV-2.

## 5. Conclusions

Many studies prove the safety and efficacy of G2-S16 polyanionic carbosilane dendrimer not only in vitro but also in vivo. Our work represents a paramount step further in a new context of the effect of G2-S16 dendrimer that showed the efficacy of this G2-S16 dendrimer against HSV-2 infection in the presence of semen. We also characterized the BALB/c mice vaginal microbiome revealing a clear dominance of *Pseudomonas*. In HSV-2 BALB/c mice vaginal infection, a significant shift in the composition of the microbiome and a high heterogeneity were observed. It has previously demonstrated that G2-S16 dendrimer is a safe and available microbicide candidate that does not cause BV. G2-S16 dendrimer vaginally applied in BALB/c mice before HSV-2 infection prevents the microbiome shift observed in the absence of the G2-S16 dendrimer. This work proves that G2-S16 dendrimer could be taken to human preclinical trials to assess its safety in women.

This work is the first to our knowledge to characterize the murine vaginal microbiota throughout estrus using 16S rRNA sequencing. We further show the influence of endogenous flora on successful colonization and by a human pathogen. This work underscores the importance of continuing to assess the native murine flora in models of human vaginal pathogens.

## Supplementary Materials

The following are available online at ’https://www.mdpi.com/1999-4923/12/6/515/s1, Figure S1: Microbiome composition of sample “R0” (five months) compared to samples analyzed in this experiment. Figure S2. Recovery of Staphylococcus sciuri genome from assembled metagenomic contigs of sample C1d8. Table S1. Summary metagenomic sequencing data. Table S2. Relative abundance of main microbes in the different samples. Table S3. Two ways PERMANOVA analysis of microbial composition. Table S4. One-way ANOVA and Tukey test.

## Abbreviations

STIs	Sexual transmission infections
BV	Bacterial vaginosis
TFV	Tenofovir
HSV-2	Herpes simplex virus type 2
HIV-1	Human immunodeficiency virus type 1
BLT	Bone-liver-thymus
DMEM	Dulbecco’s Modified Eagle’s Medium
FBS	Fetal bovine serum
IgG	Human immunoglobulin G
PFU	Plaque-forming unit
MOI	Multiplicity of infection
PBS	Phosphate buffer saline
HEC	Hydroxyethyl-cellulose
NT	Nontreated
SP	Seminal plasma
PCoA	Principal coordinate analysis
HCV	Hepatitis C virus
