# Supplementary material for: Cationic Dendrimer G2-S16 Inhibits Herpes Simplex Type 2 Infection and Protects Mice Vaginal Microbiome
Source: Pharmaceutics. 2020 Jun 4;12(6):515. doi: 10.3390/pharmaceutics12060515 (PMC7356682; doi:10.3390/pharmaceutics12060515)
Supplement: Supplementary file 1 [file pharmaceutics-12-00515-s001.pdf]

pharmaceutics

Article

Supplementary Materials: Cationic Dendrimer G2-S16 Inhibits Herpes Simplex Type 2 Infection and Protects Mice Vaginal Microbiome

Carlos Guerrero-Beltrán, Inmaculada Garcia-Heredia, Rafael Ceña-Diez, Ignacio Rodriguez-Izquierdo, María Jesús Serramía, Francisco Martinez-Hernandez, Mónica Lluesma-Gomez, Manuel Martinez-Garcia and María Ángeles Muñoz-Fernández

Table of Contents

Supplementary Figures

Supplementary Figure S1. Microbiome composition of sample "R0" (five months) compared to samples analyzed in this experiment

Supplementary Figure S2. Recovery of *Staphylococcus sciuri* genome from assembled metagenomic contigs of sample C1d8

Supplementary Tables

Supplementary Table S1. Summary metagenomic sequencing data.

Supplementary Table S2. Relative abundance of main microbes in the different samples.

Supplementary Table S3. Two way PERMANOVA analysis of microbial composition.

Supplementary Table S4. One-way ANOVA and Tukey test.

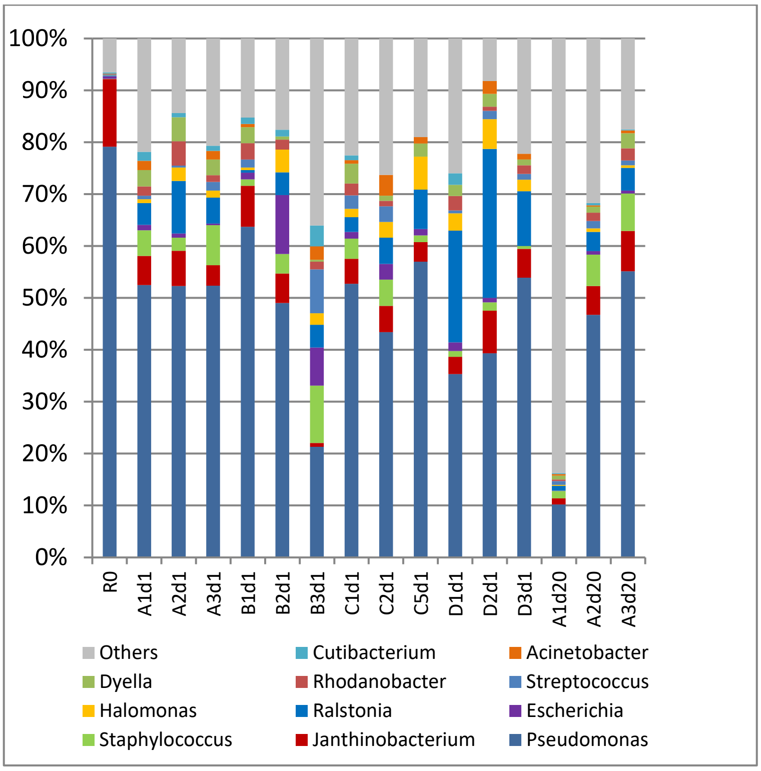

Supplementary Figure S1. Microbial composition of vagina of sample "R0" compared to samples analyzed in this experiment. Sample R0 was independently taken five months before this experiment.

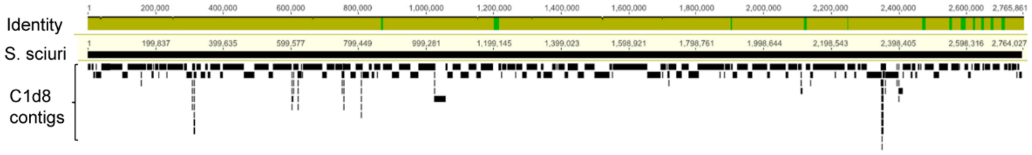

Supplementary Figure S2. Recovery of *Staphylococcus sciuri* genome from assembled metagenomic contigs of sample C1d8. Comparison using BLASTn of the contigs larger than 10Kb from sample C1d8 with *S. sciuri* genome. Plot done with Geneious bioinformatic program.

Supplementary Tables

Supplementary Table S1. Summary metagenomic sequencing data.

	Initial Data			After First Cleaning (Bmtagger)		After Trimming (Trimmomatic)		After Second Cleaning (BLASTn)	
	num. of Reads	num. of nucleotides	median length read (bp)	num. of Reads	num. of nucleotides	num. of Reads	num. of nucleotides	num. of Reads	num. of nucleotides
A1d1	40971070	6137751647	149.81	632120	94849091	532564	44779289	10078	1232015
A1d20	47813710	6957876679	145.52	667198	97355013	564483	52193370	14011	1836146
A2d1	33286120	4984533842	149.75	637444	95650948	536444	43783351	9781	1172576
A2d20	38326326	5723908572	149.35	684934	102562306	598760	53001312	11669	1433343
A3d1	39865634	5956551360	149.42	645978	96743204	559256	46823182	10886	1323186
A3d20	39990032	5947670504	148.73	658980	98297891	558682	46181836	10414	1279430
B1d1	31076726	4659105730	149.92	600958	90275475	510455	41079542	10616	1291817
B1d20	35211468	5270955802	149.69	553254	82931004	478926	42933785	15066	1997489
B2d1	30387910	4555994873	149.93	610192	91672991	517579	41404819	9385	1108573
B2d20	33409330	4982750391	149.14	552672	82629900	470578	39362409	9136	1130908
B3d1	36575224	5478702142	149.79	703748	105623120	594677	48367108	10794	1294625
B3d20	45215346	6776922230	149.88	835950	125526308	704146	57461533	13470	1642873
C1d1	42635580	6357409590	149.11	716868	107217590	597286	50580027	11306	1396272
C1d8	48386542	7250727828	149.85	1987156	298718623	1809713	205234684	890705	131141393
C2d1	13045562	1951154283	149.56	390914	58735645	349230	27602975	8533	952571
C2d3	2129986	317571217	149.1	85310	12811411	74506	5439829	1341	147051
C5d1	15406996	2303928308	149.54	528508	79403924	451581	33629568	8019	900107
C5d10	15609598	2319232979	148.58	264450	39332366	232008	20606215	9617	1323203
D1d1	33370322	4981007599	149.26	647100	96938334	552552	45135901	10190	1195788
D1d20	35684786	5351194397	149.96	609468	91565179	522486	43290277	10059	1220627
D2d1	18361802	2736194673	149.02	645936	96954671	559422	40503645	9816	1095813
D2d20	34372814	5152165259	149.89	597774	89762807	510615	42078656	9983	1221989
D3d1	34852858	5219392703	149.76	604104	90661132	520229	41590878	9046	1070611
D3d20	37864286	5675601443	149.89	659586	99056548	570981	46920003	12085	1471112

Supplementary Table S2. Relative abundance of main microbes in the different samples.

	Pseudomonas (%)	Janthinobacterium (%)	Staphylococcus (%)	Escherichia (%)	Ralstonia (%)	Halomonas (%)	Streptococcus (%)	Rhodanobacter (%)	Dyella (%)	Acinetobacter (%)	Cutibacterium (%)	Others (%)
A1d1	52.46	5.63	4.93	1.06	4.23	0.7	0.7	1.76	3.17	1.76	1.76	21.83
A2d1	52.32	6.75	2.53	0.84	10.13	2.53	0.42	4.64	4.64	0	0.84	14.35
A3d1	52.33	4	7.67	0.33	5	1.33	1.67	1.33	3	1.67	1	20.67
B1d1	63.7	7.93	1.2	1.32	0.48	0.48	1.56	3.13	3.13	0.6	1.2	15.26
B2d1	49.06	5.66	3.77	11.32	4.4	4.4	0	1.89	0.63	0	1.26	17.61
B3d1	21.32	0.74	11.03	7.35	4.41	2.21	8.46	1.47	0.37	2.57	4.04	36.03
C1d1	52.73	4.82	3.86	1.29	2.89	1.61	2.57	2.25	3.86	0.64	0.96	22.51
C2d1	43.43	5.05	5.05	3.03	5.05	3.03	3.03	1.01	1.01	4.04	0	26.26
C5d1	56.96	3.8	1.27	1.27	7.59	6.33	0	0	2.53	1.27	0	18.99
D1d1	35.36	3.31	1.1	1.66	21.55	3.31	0.55	2.76	2.21	0	2.21	25.97
D2d1	39.34	8.2	1.64	0.82	28.69	5.74	1.64	0.82	2.46	2.46	0	8.2
D3d1	53.89	5.56	0.56	0	10.56	2.22	1.11	1.67	1.11	1.11	0	22.22
A1d20	10.24	1.14	1.44	0.04	0.92	0.22	0.7	0.35	0.7	0.31	0.22	83.73
A2d20	46.76	5.56	6.02	0.69	3.7	0.69	1.39	1.62	1.16	0.23	0.46	31.71
A3d20	55.15	7.78	7.23	0.54	4.34	0.54	0.9	2.35	2.89	0.54	0.18	17.54
B1d20	63.73	8.9	0.29	0.29	0.14	0.23	0.43	2.56	2.48	0.12	0.55	20.28
B2d20	45.94	4.42	3.89	4.06	1.24	0.35	3.53	1.59	2.3	0.71	2.65	29.33
B3d20	52.37	8.32	0.9	2.18	2.94	0.64	0.64	2.43	3.33	0.51	0	25.74
C1d8	0.53	0.07	98.84	0.01	0	0	0.09	0.03	0.03	0	0	0.4
C2d3	23.08	7.69	23.08	30.77	0	0	0	0	0	0	0	15.38
C5d10	4.39	0.04	4.1	1.9	0.07	0.15	3.43	0	0	30.65	2.46	52.81
D1d20	52.41	5.17	11.03	0.69	6.21	2.76	0.34	0.34	2.76	1.38	0.69	16.21
D2d20	63.88	9.79	0.48	0.48	4.65	0.32	0.16	2.09	2.09	0.96	0.64	14.45
D3d20	54.11	8.68	0.56	0.45	4.62	0.56	0.23	3.61	2.93	7.55	0.23	16.46

Supplementary Table S3. Two way PERMANOVA analysis of microbial composition.

**> permanova**						
Call:						
adonis(formula = Y ~ infection * treatment. data = datos. permutations = 4999)						
Permutation: free<br>Number of permutations: 4999						
Terms added sequentially (first to last)						
	Df	SumsOfSqs	MeanSqs	F.Model	R2	Pr(>F)
infection	1	0.3284	0.32841	2.854	0.09936	0.0216 *
treatment	1	0.3401	0.34011	2.9556	0.1029	0.018 *
infection:treatment	1	0.3354	0.33536	2.9144	0.10146	0.0156 *
Residuals	20	2.3014	0.11507	0.69628		
Total	23	3.3053	1			
Signif. codes: 0 ‘***’ 0.001 ‘**’ 0.01 ‘*’ 0.05 ‘.’ 0.1 ‘ ’ 1						

Supplementary Table S4. One-way ANOVA and Tukey test.

Analysis of Variance Table

Response: Distances

	Df	Sum Sq	Mean Sq	Fvalue	Pr(>F)
Groups	3	0.24056	0.080188	4.1003	0.02023 *
Residuals	20	0.39113	0.019557		

Signif. codes: 0 '***' 0.001 '**' 0.01 '*' 0.05 '.' 0.1 ' ' 1

Tukey multiple comparisons of means

95% family-wise confidence level

Fit: aov(formula = distances ~ group. data = df)

$group

	diff	upr	lwr	p adj
group NT-group G2-S16	0.10461201	-0.14294143	0.3521655	0.6442643
group G2-S16+HSV-2- group G2-S16	-0.02736453	-0.34695465	0.2922256	0.9949983
group HSV-2- group G2-S16	0.33228754	0.01269742	0.6518777	0.0397527
group G2-S16+HSV-2- group NT	-0.13197654	-0.37952999	0.1155769	0.4604161
group HSV-2- group NT	0.22767553	-0.01987791	0.475229	0.0783806
group HSV-2-group G2-S16+HSV-2	0.35965207	0.04006195	0.6792422	0.0239395

Supplementary Table S5. One-way ANOVA and Tukey test.

Analysis of Variance Table

	Df	Sum Sq	Mean Sq	F value	Pr(>F)	
infection	1	3.784	3.784	8.145	0.00981	**
treatment	1	0.205	0.205	0.442	0.51386	
infection:treatment	1	0.836	0.836	1.798	0.19493	
Residuals	20	9.292	0.465			

Signif. codes: 0 '***' 0.001 '**' 0.01 '*' 0.05 '.' 0.1 ' ' 1

Tukey multiple comparisons of means

95% family-wise confidence level

Fit: aov(formula = shannon ~ type + treatment + type * treatment)

$type				
	diff	lwr	upr	p adj
HSV-2-healthy	-0.9169932	-1.587235	-0.246751	0.0098113

$treatment				
	diff	lwr	upr	p adj
+ G2-S16-- G2-S16	0.2013461	-0.4688961	0.8715884	0.5379829

$`type:treatment`					
		diff	lwr	upr	p adj
HSV-2:-G2-S16-healthy:-G2-S16	group HSV-2 - group NT	-1.346517	-2.553092	-0.1399419	0.0253253
healthy:+G2-S16-healthy:-G2-S16	group G2-S16 - group NT	-0.1275046	-1.3340796	1.0790705	0.9907173
HSV-2:+G2-S16-healthy:-G2-S16	group G2-S16+HSV-2 - group NT	-0.5299709	-1.736546	0.6766041	0.6161502
healthy:+G2-S16-HSV-2:-G2-S16	group G2-S16 - group HSV-2	1.2190124	-0.3386693	2.7766941	0.1600785
HSV-2:+G2-S16-HSV-2:-G2-S16	group G2-S16+HSV-2 - group HSV-2	0.8165461	-0.7411356	2.3742278	0.4746646
HSV-2:+G2-S16-healthy:+G2-S16	group G2-S16+HSV-2 - group G2-S16	-0.4024664	-1.9601481	1.1552153	0.8866865
